# Intrinsic disorder is essential for Cas9 inhibition of anti-CRISPR AcrIIA5

**DOI:** 10.1093/nar/gkaa512

**Published:** 2020-06-16

**Authors:** So Young An, Donghyun Ka, Iktae Kim, Eun-Hee Kim, Nak-Kyoon Kim, Euiyoung Bae, Jeong-Yong Suh

**Affiliations:** Department of Agricultural Biotechnology and Research Institute of Agriculture and Life Sciences, Seoul National University, Seoul 08826, South Korea; Department of Agricultural Biotechnology and Research Institute of Agriculture and Life Sciences, Seoul National University, Seoul 08826, South Korea; Department of Agricultural Biotechnology and Research Institute of Agriculture and Life Sciences, Seoul National University, Seoul 08826, South Korea; Protein Structure Research Team, Korea Basic Science Institute, 162 Yeongudanji-Ro, Ochang, Chungbuk 28119, South Korea; Advanced Analysis Center, Korea Institute of Science and Technology, Seoul 02792, South Korea; Department of Agricultural Biotechnology and Research Institute of Agriculture and Life Sciences, Seoul National University, Seoul 08826, South Korea; Department of Agricultural Biotechnology and Research Institute of Agriculture and Life Sciences, Seoul National University, Seoul 08826, South Korea; Institute for Biomedical Sciences, Shinshu University, Minamiminowa, Nagano 399-4598, Japan

## Abstract

Clustered regularly interspaced short palindromic repeats (CRISPRs) and CRISPR-associated (Cas) proteins provide adaptive immunity to prokaryotes against invading phages and plasmids. As a countermeasure, phages have evolved anti-CRISPR (Acr) proteins that neutralize the CRISPR immunity. AcrIIA5, isolated from a virulent phage of *Streptococcus thermophilus*, strongly inhibits diverse Cas9 homologs, but the molecular mechanism underlying the Cas9 inhibition remains unknown. Here, we report the solution structure of AcrIIA5, which features a novel α/β fold connected to an N-terminal intrinsically disordered region (IDR). Remarkably, truncation of the N-terminal IDR abrogates the inhibitory activity against Cas9, revealing that the IDR is essential for Cas9 inhibition by AcrIIA5. Progressive truncations and mutations of the IDR illustrate that the disordered region not only modulates the association between AcrIIA5 and Cas9–sgRNA, but also alters the catalytic efficiency of the inhibitory complex. The length of IDR is critical for the Cas9–sgRNA recognition by AcrIIA5, whereas the charge content of IDR dictates the inhibitory activity. Conformational plasticity of IDR may be linked to the broad-spectrum inhibition of Cas9 homologs by AcrIIA5. Identification of the IDR as the main determinant for Cas9 inhibition expands the inventory of phage anti-CRISPR mechanisms.

## INTRODUCTION

Genomes of prokaryotic organisms often contain clustered regularly interspaced short palindromic repeat (CRISPR) loci that are composed of repeated DNA sequences alternating with variable sequences of viral origin (1). The CRISPR region is transcribed and processed into CRISPR RNAs (crRNAs) that associate with CRISPR-associated (Cas) proteins to form an RNA-guided ribonuclease complex (2). When viruses or foreign plasmids invade bacteria or archaea, the ribonuclease complex effectively destroys foreign DNA or RNA sequences targeted by crRNA. The CRISPR–Cas system acquires foreign DNA fragments from invading genetic materials, stores past infection records in chronological order, and retrieves the stored information to find and cleave matching nucleic acids, which resembles the adaptive immune system in vertebrates (3).

It is now well established that CRISPR–Cas constitutes one of the major defense mechanisms in prokaryotes against invading phages and plasmids (4,5). The CRISPR–Cas system is divided into two classes according to the composition of the interference complex: multi-Cas proteins participate in the interference complex for class 1, and a single effector protein is fully functional for class 2 (6). The class 1 CRISPR–Cas system is categorized into types I, III and IV, and the class 2 into types II, V and VI depending on Cas proteins involved and nucleic acids targeted. The six CRISPR–Cas types are further divided into dozens of subtypes according to the signature *Cas* gene structures (7,8).

Bacteria and bacteriophages have long evolved weapons for defense and invasion as hosts and parasites, respectively (9,10). Since CRISPR function was first annotated as the bacterial defense system, counter-defense mechanisms of phages were anticipated, and the first anti-CRISPR (Acr) proteins were discovered in phages infecting *Pseudomonas aeruginosa* with the type I-F CRISPR–Cas system (11). The search for new Acr proteins rapidly gained momentum, expanding the repertoire of their targets in the type I CRISPR–Cas system (12,13). Acr proteins against the type II CRISPR–Cas system, which effectively disabled the nuclease activity of host Cas9 proteins, were later discovered in mobile genetic elements of *Neisseria meningitidis* (type II-C) and prophages of *Listeria monocytogenes* (type II-A) (14,15).

Structural investigation of type II Acr proteins revealed unique folds and distinct mechanisms for Cas9 inhibition. AcrIIA1 exhibits bi-functional modality, with the N-terminal domain performing auto-repression and the C-terminal domain inhibiting Cas9 (16,17). AcrIIA2 and AcrIIA4 associate with the protospacer adjacent motif binding site of Cas9, and prevent target DNA recognition (18–22). AcrIIA6 inhibits target DNA binding to Cas9 via an allosteric mechanism (23,24). AcrIIC1 blocks the active site of the HNH nuclease domain of Cas9, whereas AcrIIC3 associates with the HNH and REC domains of Cas9 to induce a functionally incompetent Cas9 dimer (25–28). Lastly, AcrIIC2 associates with the bridge helix of Cas9, competing with guide RNA for Cas9 binding (26,29).

AcrIIA5 was discovered in a virulent phage infecting *Streptococcus thermophilus* and inhibits *S. thermophilus* Cas9 as well as *Streptococcus pyogenes* Cas9, a widely used nuclease for genome editing (30). AcrIIA5 abolished *S. pyogenes* Cas9 activity in CRISPR-immunized bacterial cultures in a phage challenge assay, but the molecular mechanism underlying Cas9 inhibition remains unclear. Here, we show that AcrIIA5 adopts a novel α/β fold preceded by an intrinsically disordered region (IDR). Remarkably, the IDR is crucial for Cas9 inhibition, such that truncation of the IDR led to a complete loss of the Acr activity of AcrIIA5. The length and charge of IDRs modulated AcrIIA5 binding to Cas9 and concomitant inhibition of the nuclease activity, illustrating a unique role of the IDR in Cas9 inhibition.

## MATERIALS AND METHODS

### Cloning, expression and purification of AcrIIA5 and mutants

The synthetic *acrIIA5* and mutant genes were cloned into a pET28 vector with an N-terminal His_6_-tag and a maltose-binding protein (MBP) tag. The cloned vectors were transformed into the *Escherichia coli* strain BL21Star(DE3) (Invitrogen). Cells were grown in lysogeny broth or minimal medium supplemented with ^15^NH_4_ and ^13^C_6_-glucose as the nitrogen and carbon sources, respectively, in H_2_O. When *A*_600_ reached 0.6–0.8, cells were treated with 1 mM isopropyl β-d-1-thiogalactopyranoside for 20 h at 18°C, and then harvested by centrifugation. Harvested cells were resuspended in 20 mM Tris–HCl, pH 7.4, 500 mM NaCl, 5% glycerol, and 5 mM β-mercaptoethanol (Buffer A) with 1 mM phenylmethyl sulfonyl fluoride (PMSF), lysed by Emulsiflex (Avestin), and centrifuged at 40 000 × g for 30 min. Supernatants were loaded onto a HisTrap column (GE Healthcare) and eluted with a 0–500 mM imidazole gradient. The His_6_- and MBP-tags were cleaved using a tobacco etch virus protease in Buffer A. The untagged proteins were loaded onto an SP column (GE Healthcare) and eluted with a 0−1 M NaCl gradient. The proteins were finally purified on a Superdex 75 26/60 column (GE Healthcare) equilibrated with Buffer A.

### Size exclusion chromatography

Analytical size exclusion chromatography of AcrIIA5 was performed on a Superdex 75 10/300 GL column (GE Healthcare). The column was equilibrated with Buffer A, and the sample was eluted isocratically at a flow rate of 1 ml/min.

### NMR spectroscopy

NMR spectra were collected at 25°C on Bruker AVANCE III 600, 700, 800 and 900 MHz spectrometers equipped with a *z*-shielded gradient triple resonance cryoprobe. NMR spectra were processed using the NMRPipe program (31) and analyzed using the PIPP/CAPP/STAPP (32), NMRView (33) and NMRFAM-SPARKY (34) programs. Sequential assignment of ^13^C/^15^N-labeled AcrIIA5 protein was performed using the 2D ^15^N-HSQC spectrum and 3D triple resonance through-bond scalar correlation experiments including 3D HNCO, HNCACO, HNCA, HN(CO)CA, HNCACB, CBCA(CO)NH experiments. ^1^H–^15^N heteronuclear NOE measurements were acquired using 3 s of 120° ^1^H pulses separated by 5 ms intervals using a previously employed pulse program (35).

### Structure calculation

Interproton distance restraints were derived from the NOE spectra and classified into distance ranges according to the peak intensity. φ/ψ torsion angle restraints were derived from backbone chemical shifts using the program TALOS+ (36). Structures were calculated by simulated annealing in torsion angle space using the Xplor-NIH program (37). The target function for simulated annealing included covalent geometry, a quadratic van der Waals repulsion potential, square-well potentials for interproton distance and torsion angle restraints, hydrogen bonding, harmonic potentials for ^13^Cα/^13^Cβ chemical shift restraints (38), and a multidimensional torsion angle database potential of mean force (39). Structures were displayed using PyMOL software (The PyMOL Molecular Graphics System, Version 2.0 Schrödinger, LLC.).

### Preparation of Cas9 and Cas12a

*S. pyogenes* Cas9 was cloned into a pET28 vector with an N-terminal His_6_-tag. The cloned vector was transformed into the *Escherichia coli* strain BL21Star(DE3) (Invitrogen). Cells were grown in lysogeny broth, induced by 1 mM isopropyl β-d-1-thiogalactopyranoside for 20 h at 18°C, and harvested by centrifugation as described above. The harvested cells were resuspended in 20 mM Tris–HCl, pH 7.4, 300 mM NaCl, 20 mM imidazole, 10% glycerol and 5 mM β-mercaptoethanol (Buffer B) with 1 mM PMSF, lysed using Emulsiflex, and centrifuged at 40 000 × g for 30 min. The supernatant was loaded onto the HisTrap column and eluted with a 0–500 mM imidazole gradient. Cas9 was further purified using an SP column with a 0−1 M NaCl gradient, and then using a Superdex 200 26/60 column (GE Healthcare) equilibrated with Buffer B. *Lachnospiraceae bacterium* ND2006 Cas12a was purchased from New England Biolabs (Cat # M0653S).

### sgRNA preparation

DNA encoding a minimal T7 promoter upstream of an sgRNA of *S. pyogenes* Cas9 (with a random sequence without targeting sites in *E. coli*: 5′-GGAAATTAGGTGCGCTTGGCGTTTTAGAGCTAGAAATAGCAAGTTAAAATAAGGCTAGTCCGTTATCAACTTGAAAAAGTGGCACCGAGTCGGTGCTT-3′) and an sgRNA of *L. bacterium* Cas12a (with a random sequence without targeting sites in *E. coli*: 5′- TAATTTCTACTAAGTGTAGATGGAAATTAGGTGCGCTTGGC-3′) was synthesized by Bioneer. The DNA template for RNA transcription was prepared by using the Gigaprep kit (ZYMO RESEARCH). The sgRNA was prepared in vitro by mixing rNTPs, MgCl_2_, T7 RNA polymerase (P266L mutant), inorganic pyrophosphatase (IPP), and the DNA template in the transcription buffer. After 6 h of transcription at 37°C, synthesized RNA was precipitated by ethanol treatment overnight, dissolved in D_2_O, and purified using 12% denaturing PAGE (19:1 cross-linking ratio) by electro-elution (Elutrap, Whatman; GE Healthcare). Purified RNA was washed using 1.5 M NaCl, and finally prepared in water using Amicon Ultra Centrifugal Filters (Merck Millipore).

### *In vitro* DNA cleavage assay

Cas9 and sgRNA (each at 500 nM) were mixed in 20 mM Tris–HCl, pH 7.5, 150 mM KCl, 5 mM MgCl_2_, 1 mM dithiothreitol (DTT) and 5% (v/v) glycerol at 37°C for 5 min. AcrIIA5 was added to the Cas9–sgRNA complex at 250–3000 nM for 5 min, and the linearized 20 nM DNA substrate was finally added to the mixture and incubated at 37°C for 15 min. The reaction products were treated with proteinase K at 50°C for 15 min to stop the reaction. The DNA reactions were mixed with the Loading Star dye (Dynebio) and analyzed on a 1% agarose gel.

### *In vitro* RNA cleavage assays

Cas9 (200 nM) and sgRNA (400 nM) were mixed in 20 mM Tris–HCl, pH 7.5, 150 mM KCl, 5 mM MgCl_2_, 1 mM DTT and 5% (v/v) glycerol at 25°C for 20 min. AcrIIA5 (4 mM) was added to the Cas9–sgRNA complex, and incubated at 25°C for 40 min. The reaction products were treated with proteinase K at 50°C for 15 min to stop the reaction. sgRNA was analyzed by 10% urea polyacrylamide gel electrophoresis and stained by SYBR Gold (Thermo Fisher Scientific).

### Electrophoretic mobility shift assay

sgRNA (200 nM) with or without Cas9 (400 nM) was mixed with AcrIIA5 at 20–2000 nM in 20 mM Tris–HCl, pH 7.5, 150 mM KCl, 5 mM MgCl_2_, 1 mM DTT and 5% (v/v) glycerol at 25°C for 40 min. sgRNAs were analyzed by 6% polyacrylamide gel electrophoresis and stained by SYBR Gold.

## RESULTS

### AcrIIA5 adopts a novel fold with an IDR

**Figure 1.**
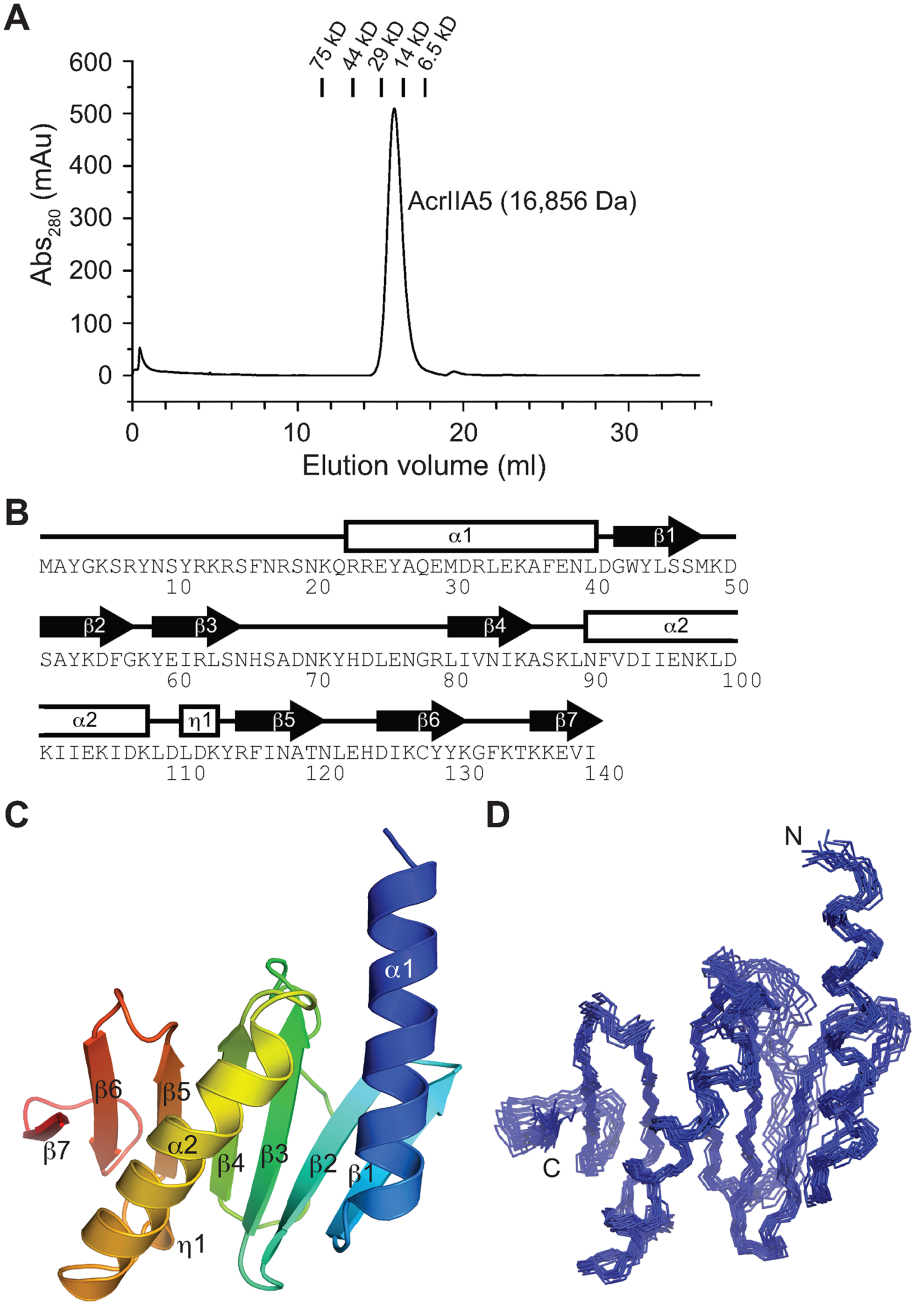
The chromatogram and the solution structure of AcrIIA5. (**A**) The size exclusion chromatogram of AcrIIA5 using a Superdex 75 30/100 GL column. The elution profiles of standard marker proteins are shown on top of the chromatogram as a reference, and the calculated molecular weight of AcrIIA5 is annotated. (**B**) Schematic representation of the secondary structure of AcrIIA5 shown above the amino acid sequence. (**C**) The lowest-energy solution structure of AcrIIA5 in a cartoon diagram and rainbow color scheme. The disordered N-terminal residues (a.a. 1–22) are omitted for visual clarity, and the secondary structures are annotated. (**D**) Superimposition of the backbone atoms of the final 20 simulated annealing structures of AcrIIA5.

AcrIIA5 (a.a. 1–140) is a basic protein with a theoretical isoelectric point of 9.4, and elutes as a monomer in solution by size exclusion chromatography (Figure 1A). We could express recombinant AcrIIA5 with an N-terminal maltose-binding protein tag, but AcrIIA5 in the absence of the tag tended to aggregate during purification due to its low solubility at low ionic strength (150 mM NaCl). The solubility dramatically increased at high ionic strength, such that >0.5 mM AcrIIA5 could be prepared at 500 mM NaCl. We determined the solution structure of AcrIIA5 at 500 mM NaCl, based on 2032 experimental NOE restraints from three-dimensional ^13^C-separated NOESY and ^15^N-separated NOESY experiments (Table 1). Backbone and side chain ^1^H, ^13^C and ^15^N resonances were assigned using a suite of heteronuclear correlation NMR spectroscopy. Backbone amide resonances of AcrIIA5 were annotated in the 2D ^1^H–^15^N heteronuclear single quantum correlation (HSQC) spectrum (Supplementary Figure S1A).

**Table 1.** Restraints and structural statistics of AcrIIA5

Experimental restraints	<SA>^a^
Nonredundant NOEs	2,032
Intra-residue NOEs	878
Inter-residue NOEs	1154
Sequential (| *i* – *j* | = 1)	573
Medium-range (1 < | *i* – *j* | ≤ 4)	263
Long-range (| *i* – *j* | > 4)	318
Dihedral angles, φ/ψ	98/98
Hydrogen bonds	58
Total number of restraints	2286 (16.3 per residue)
Rms deviation from experimental restraints	
Distances (Å) (2032)	0.016 ± 0.001
Torsion angles (°) (196)	0.487 ± 0.038
Rms deviation from idealized covalent geometry	
Bonds (Å)	0.001 ± 0
Angles (°)	0.362 ± 0.003
Impropers (°)	0.237 ± 0.006
Coordinate precision (Å)^b^	
Backbone	0.84 ± 0.10
Heavy atoms	1.59 ± 0.11
Ramachandran statistics (%)^b^	
Favored regions	95 ± 1
Allowed regions	3 ± 1
Outliers	2 ± 1

^a^For the ensemble of the final 20 simulated annealing structures.

^b^Residues 23−140, excluding disordered N-terminal residues 1−22 and loop residues 66−79.

AcrIIA5 comprises seven β-strands and two α-helices, preceded by an extended N-terminal disordered region (Figure 1B). Two antiparallel β-sheets of β1–β2–β3 and β5–β6–β7 are bridged by a β4 strand that forms a parallel β3–β4–β5 sheet, and the α1 and α2 helices sit on the same side of the β-sheet (Figure 1C). Overall secondary structures form a well-defined fold except for a long loop between strands β3 and β4 (Figure 1D). A search for structural homologs of AcrIIA5 using the DALI program did not return a similar fold, indicating that AcrIIA5 adopts a novel fold (40). Previously, AcrIIA5 was predicted to contain a coiled-coil motif (30). The predicted regions indeed corresponded to α1 and α2 helices, but they did not form a coiled-coil structure.

**Figure 2.**
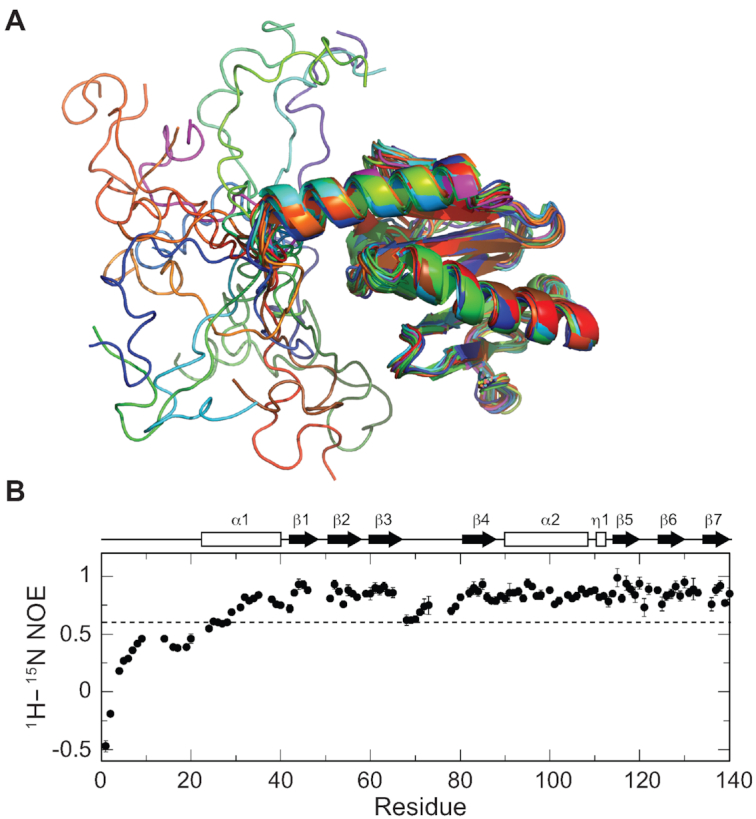
Characterization of the N-terminal IDR of AcrIIA5. (**A**) Illustration of the N-terminal IDR of AcrIIA5. The ensemble of 20 NMR structures was superimposed using the secondary structural region, and presented in a cartoon diagram. (**B**) ^1^H–^15^N heteronuclear NOE data as a function of the residue number of AcrIIA5. A dashed line denotes the heteronuclear NOE value of 0.6, and secondary structures are shown above the NOE data.

We note that the N-terminal 22 residues of AcrIIA5 display largely disordered backbone conformations (Figure 2A). Since Acr proteins generally adopt a compact fold without extended tail regions, we further investigated the IDR of AcrIIA5. When 20 residues of AcrIIA5 were removed from the N-terminus (AcrIIA5_Δ20_), the protein still maintained the backbone fold of the full-length AcrIIA5. Superimposition of ^1^H–^15^N HSQC spectra of the N-terminally truncated and the full-length AcrIIA5 proteins revealed that chemical shifts of backbone amide resonances were mostly identical for the folded region (Supplementary Figure S1B). The absence of chemical shift changes in the folded region indicated that N-terminal IDR does not interact with the folded region. In addition, the N-terminal tail region exhibited a narrow dispersion of backbone chemical shifts, which is typically observed in unfolded proteins (Supplementary Figure S1B). Furthermore, the ^1^H–^15^N heteronuclear NOE values clearly indicated that the N-terminal residues were highly mobile (Figure 2B). Heteronuclear NOEs are sensitive to fast internal dynamics, and thus can discriminate flexible loop and tail regions from rigid secondary structures. Residues in the folded region of AcrIIA5 showed large ^1^H–^15^N heteronuclear NOE values (>0.8), whereas the N-terminal disordered region exhibited significantly reduced NOE values (<0.6), a signature of conformational flexibility (Figure 2B). In addition, the N-terminal half of the α1 helix and the β3–β4 loop region exhibited increased mobility in their backbone conformations. Taken together, AcrIIA5 features an IDR at its N-terminus, which is highly mobile independent of the structured region.

### N-terminal disorder is essential for Cas9 inhibition by AcrIIA5

**Figure 3.**
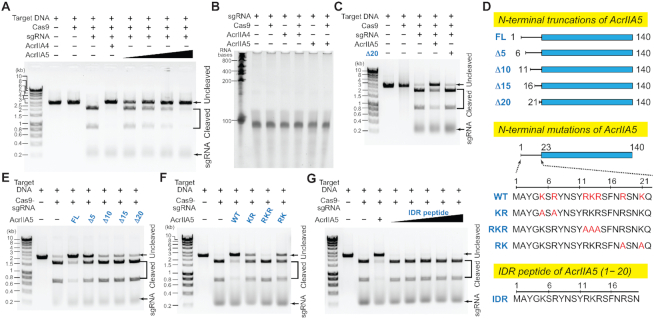
Impact of the IDR length and charge on Cas9 inhibition by AcrIIA5. (**A**) DNA cleavage assay of *S. pyogenes* Cas9−sgRNA (0.5 μM) in the presence of AcrIIA4 (3 μM) and AcrIIA5 (0.25, 0.5, 1 and 3 μM). (**B**) Analysis of sgRNA (0.2 μM) cleavage in the presence and absence of Cas9 (0.4 μM), AcrIIA4 (4 μM) and AcrIIA5 (4 μM) on a urea gel. (**C**) DNA cleavage assay of Cas9−sgRNA (0.5 μM) in the presence of AcrIIA5 (1 μM) or AcrIIA5_Δ20_ (1 μM). (**D**) Domain constructs of AcrIIA5 with serial truncations (top) and charge mutations of the IDR (middle), and the IDR peptide sequence (bottom). (E−G) DNA cleavage assay of Cas9−sgRNA (0.5 μM) in the presence of (**E**) AcrIIA5 with serial truncations of IDR (3 μM), (**F**) AcrIIA5 with charge mutations of IDR (3 μM), and (**G**) the isolated IDR peptide (a.a. 1–20; 0.25, 0.5, 1, 3, and 5 μM) of AcrIIA5.

It was previously shown that AcrIIA5 of virulent phages effectively inhibited both *S. thermophilus* and *S. pyogenes* Cas9 functions *in vivo*, but the molecular mechanism underlying the Cas9 inactivation was not clearly reported (30). We investigated the inhibitory mechanism of AcrIIA5 against *S. pyogenes* Cas9, which is widely used in genome editing. Purified AcrIIA5 completely inhibited target double-stranded DNA (dsDNA) cleavage of Cas9–sgRNA in the *in vitro* nuclease activity assay, demonstrating that AcrIIA5 alone is sufficient for Cas9 inhibition (Figure 3A). We used AcrIIA4 as a positive control of Cas9 inhibition in Figure 3A. AcrIIA5 did not inhibit *L. bacterium* Cas12a, another RNA-guided endonuclease from the type V-A CRISPR–Cas system (Supplementary Figure S2A).

It has recently been reported that co-expression of AcrIIA5 with Cas9 and sgRNA leads to the cleavage of sgRNA while still bound to Cas9 (41). This finding raised the possibility that AcrIIA5 could participate directly or indirectly in sgRNA cleavage for Cas9 inhibition. We examined whether AcrIIA5 possessed intrinsic ribonuclease activity. Treatment of free sgRNA or the Cas9–sgRNA complex with AcrIIA5 did not digest sgRNA, and the AcrIIA5-treated sgRNA bands remained intact in the urea gel (Figure 3B). Thus, the sgRNA cleavage observed in previously reported co-expression systems can be attributed to endogenous ribonucleases (41). Notwithstanding, the observation of sgRNA cleavage in the presence of AcrIIA5 *in vivo* suggests that AcrIIA5 possibly interferes with the Cas9–sgRNA assembly, leaving sgRNA vulnerable to cellular ribonucleases.

We employed the N-terminal truncated AcrIIA5_Δ20_ to examine whether the IDR was dispensable for Cas9 inhibition by AcrIIA5. Remarkably, AcrIIA5_Δ20_ completely lost its ability to inhibit Cas9, revealing that the N-terminal disordered region is essential for Acr activity (Figure 3C). We then progressively truncated the N-terminal region of AcrIIA5 and measured the Acr activity of the mutants (Figure 3D). Truncation of the first 5 N-terminal residues (AcrIIA5_Δ5_) showed a ∼40% reduction of Cas9 inhibition, and further truncations abolished the Acr activity (Figure 3E). Thus, the length of the IDR was important for Cas9 inhibition of AcrIIA5, such that an IDR length of >20 residues was required to maintain the maximal inhibitory activity against Cas9.

The N-terminal IDR of AcrIIA5 is rich with basic residues: 7 of its 22 residues are lysines and arginines (Figure 3D). We examined whether these positive charges in the IDR were important for Cas9 inhibition by AcrIIA5. We mutated the basic residues to alanine for charge neutralization, and prepared three mutants: K5A/R7A (AcrIIA5_KR_), R12A/K13A/R14A (AcrIIA5_RKR_), and R18A/K21A (AcrIIA5_RK_) (Figure 3D). AcrIIA5_KR_ and AcrIIA5_RK_ lost ∼50% of their inhibitory activity against Cas9, whereas AcrIIA5_RKR_ completely lost its Acr activity (Figure 3F). This result demonstrates that the positive charges in the IDR of AcrIIA5 are critical for Cas9 inhibition. In particular, the positive-charge cluster of Arg12, Lys13 and Arg14 was the most important for Cas9 inhibition, and positive charges in its vicinity further enhanced the Acr activity. We note that the truncation of the first 10 residues in the N-terminus (AcrIIA5_Δ10_) left the positive-charge cluster of Arg12/Lys13/Arg14 intact, but lost the inhibitory activity against Cas9 (Figure 3E). Thus, the IDR requires a minimal length and positive charges in order to secure the full Acr activity of AcrIIA5.

The IDR peptide alone (residues 1–20) failed to show any inhibitory activity against Cas9, indicating that the IDR functions in Cas9 inhibition only in concert with the folded region of AcrIIA5 (Figure 3G). We also examined if the IDR peptide would complement the loss-of-function AcrIIA5 mutants for Cas9 inhibition. The IDR peptide mixed with the IDR-truncated AcrIIA5_Δ20_ mutant failed to inhibit Cas9 nuclease activity (Supplementary Figure S2C). In addition, the IDR peptide mixed with AcrIIA5_RKR_, which contains the full-length IDR minus key positive charges, did not show any inhibitory activity against Cas9 (Supplementary Figure S2D). Taken together, the Cas9 inhibition of AcrIIA5 requires an IDR sequence that is covalently linked to the structured region.

### The IDR mediates direct interaction between AcrIIA5 and Cas9–sgRNA

**Figure 4.**
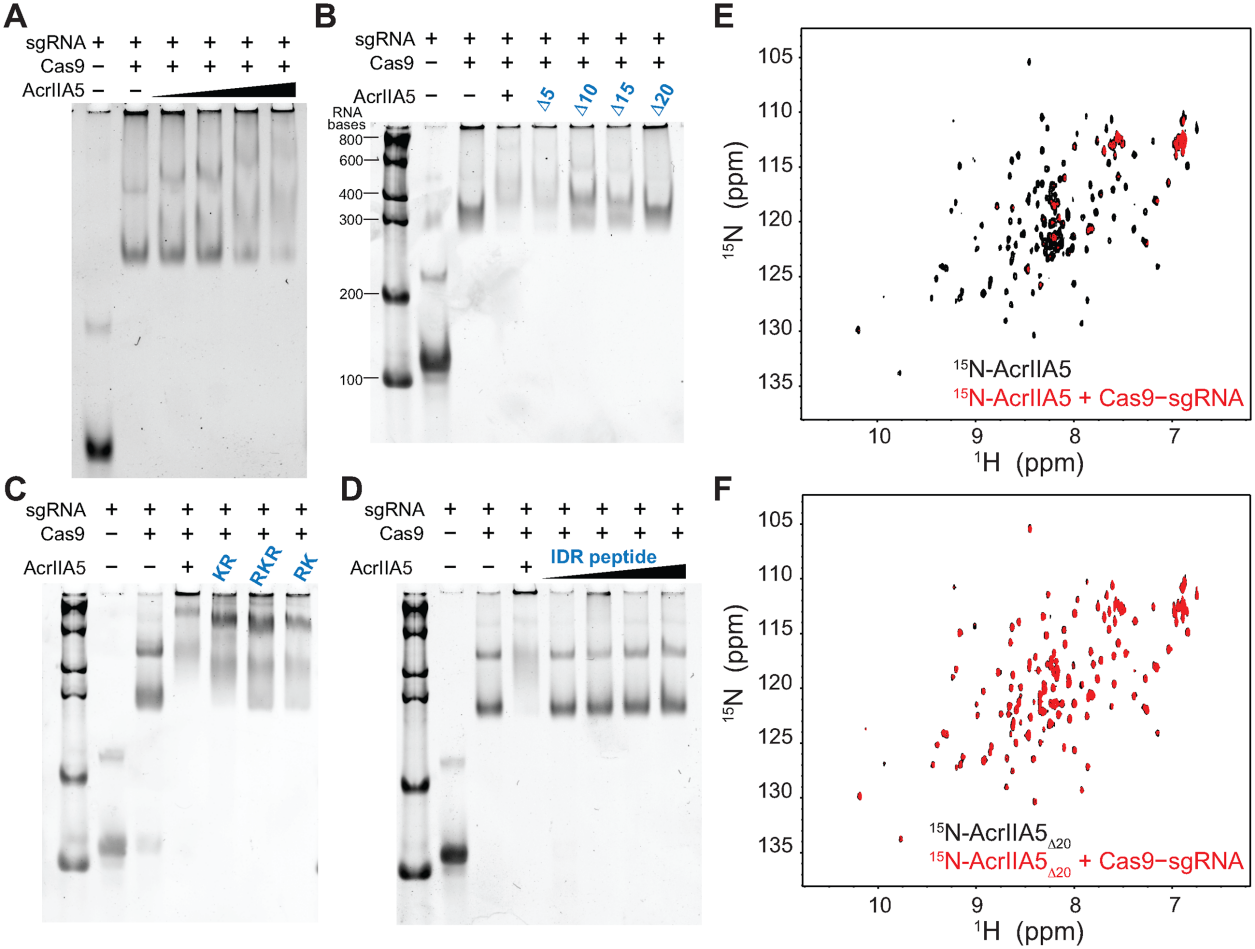
Interaction between AcrIIA5 and Cas9–sgRNA via gel shift assay and NMR spectroscopy. Changes in the electrophoretic mobility shift profiles of Cas9–sgRNA (0.2 μM) in the presence of (**A**) AcrIIA5 (0.4, 0.8, 2 and 4 μM), (**B**) AcrIIA5 (4 μM) with serial truncations of IDR, (**C**) AcrIIA5 (4 μM) with positive charge mutations of IDR and (**D**) the isolated IDR peptide (0.4, 0.8, 2 and 4 μM). 2D ^1^H–^15^N HSQC spectra of (**E**) ^15^N-AcrIIA5 and (**F**) ^15^N-AcrIIA5_Δ20_ are shown in the absence (*black*) and in the presence (*red*) of Cas9–sgRNA.

To investigate the mechanism of IDR-mediated Cas9 inhibition, we examined the interaction between AcrIIA5 and Cas9–sgRNA using an electrophoretic mobility shift assay. Titration of AcrIIA5 into Cas9–sgRNA resulted in sgRNA super-shifts owing to the ternary complex formation, demonstrating a direct interaction between AcrIIA5 and Cas9–sgRNA (Figure 4A). AcrIIA5 did not interact with *L. bacterium* Cas12a (Supplementary Figure S2B). We note that the fainter intensity of Cas9–sgRNA bands complexed with AcrIIA5 does not originate from Cas9 stability. When Cas9–sgRNA was incubated with AcrIIA5 for an extended time period, we did not observe any sign of Cas9–sgRNA degradation (Supplementary Figure S2E and F). We also note that AcrIIA5 did not induce nonspecific aggregation of Cas9–sgRNA. After we mixed AcrIIA5 with Cas9–sgRNA, and confirmed the Cas9 inhibition, we could separate AcrIIA5 from Cas9–sgRNA using the size exclusion chromatography. The purified Cas9–sgRNA from the inhibition reaction restored the nuclease activity (Supplementary Figure S2G). Thus, the decrease in band intensities may suggest a multiple binding mode or a moderate affinity interaction between Cas9–sgRNA and AcrIIA5. Next, we monitored the super-shifts of Cas9–sgRNA to examine whether the IDR would affect AcrIIA5 binding to Cas9–sgRNA. Progressive truncations of IDR gradually attenuated the interaction between AcrIIA5 and Cas9–sgRNA and AcrIIA5_Δ20_ completely lost its binding to Cas9–sgRNA (Figure 4B). Direct interaction between AcrIIA5 and Cas9–sgRNA was further supported by NMR titration experiments. When ^15^N-AcrIIA5 was titrated with Cas9–sgRNA, amide resonances of AcrIIA5 exhibited significant line-broadening, corroborating the ternary complex formation (Figure 4E). In contrast, the amide resonances of ^15^N-AcrIIA5_Δ20_ remained unchanged regardless of the presence of Cas9–sgRNA (Figure 4F). These results illustrate that the N-terminal IDR of AcrIIA5 serves as a key interface for Cas9–sgRNA to inhibit the nuclease activity. We note that partly truncated AcrIIA5 induced incremental super-shifts of Cas9–sgRNA, which did not culminate in Cas9 inhibition (Figures 3B and 4E). This finding suggests that the affinity of AcrIIA5 for Cas9–sgRNA increases in proportion to the length of the IDR, but successful Cas9 inhibition requires a full-length IDR.

Next, we examined the impact of the positive charges in the IDR on AcrIIA5 binding to Cas9–sgRNA. Positive charge mutations in AcrIIA5_KR_, AcrIIA5_RKR_ and AcrIIA5_RK_ that significantly impaired Cas9 inhibition (Figure 3F) did not abrogate the binding of AcrIIA5 to Cas9–sgRNA (Figure 4C). The charge mutants of the IDR exhibited distinct super-shift profiles of Cas9–sgRNA, indicating direct interactions (Figure 4C). This observation led us to the idea that AcrIIA5 binding to Cas9 and concomitant inhibition of the nuclease activity are dictated by different characteristics of the IDR. Specifically, the length of the IDR provides a scaffold for association between AcrIIA5 and Cas9–sgRNA, while the positive charges of the IDR serve as a regulatory switch that hinders Cas9 function upon binding. We postulate that the positive charges of the IDR may directly occlude the catalytic site of the Cas9 nuclease domain, or indirectly interferes with conformational transitions of Cas9 required for target DNA cleavage. It has recently been reported that AcrIIA5 inhibited the RuvC nuclease domain of Cas9 instead of the HNH nuclease domain, suggesting the RuvC domain as a potential target of the IDR (42). The IDR peptide alone did not show any interaction with Cas9–sgRNA (Figure 4D).

We note that AcrIIA5 interacted only with the preformed Cas9–sgRNA complex, and not with apo-Cas9. ^15^N-AcrIIA5 mixed with apo-Cas9 showed little change in the HSQC spectrum, indicating a lack of binding (Supplementary Figure S3). This is consistent with our observation that AcrIIA5 elicited the same super-shifts of Cas9–sgRNA regardless of the mixing order of Cas9, sgRNA, and AcrIIA5. Adding sgRNA to the mixture of Cas9 and AcrIIA5, or adding Cas9 to the mixture of sgRNA and AcrIIA5 induced the same super-shifts as those of Cas9–sgRNA mixed with AcrIIA5. Thus, AcrIIA5 does not interfere with sgRNA loading onto Cas9, and preferentially binds to Cas9–sgRNA over apo-Cas9. Previously, AcrIIA4 showed a similar preference for Cas9–sgRNA binding, such that AcrIIA4 bound to the protospacer adjacent motif interaction site of Cas9–sgRNA to compete with target DNA binding (43). It has been reported, however, that AcrIIA5 associated with Cas9–sgRNA regardless of the presence of target DNA (42). Taken together, AcrIIA5 selectively binds to sgRNA-loaded Cas9 and inhibits the nuclease activity without competing with target DNA binding, which is unique among Cas9 inhibitors.

### Role of the structured region of AcrIIA5 in Cas9 inhibition

**Figure 5.**
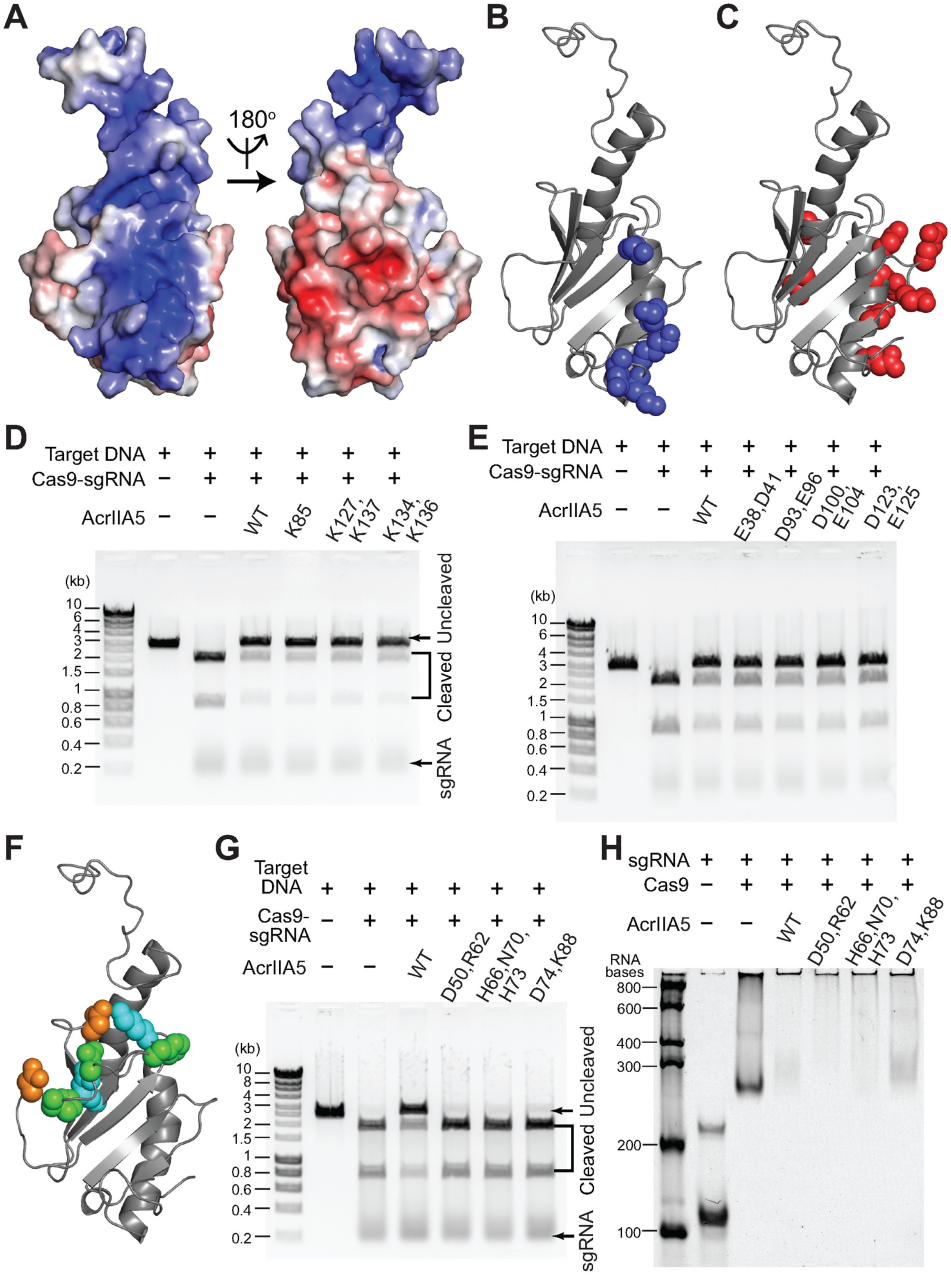
Electrostatic surface potential of AcrIIA5, and the influence of surface charge mutations on Cas9 inhibition. (**A**) Structure of AcrIIA5 with electrostatic surface potential in a surface representation for the positively- and negatively-charged surface. (**B**) Basic residues (*blue*) and (**C**) acidic residues (*red*) selected for mutagenesis are shown in a space-filling model. DNA cleavage assay of *S. pyogenes* Cas9−sgRNA (0.5 μM) in the presence of AcrIIA5 mutants (3 μM) for (**D**) basic residues and (**E**) acidic residues. (**F**) Residues that affect the Acr activity of AcrIIA5 in vivo are shown in a space-filling model: Asp50/Asp74 (*orange*), Arg62/Lys88 (*cyan*), and His66/Asn70/His73 (*green*). (**G**) DNA cleavage assay, and (H) gel shift assay of *S. pyogenes* Cas9−sgRNA against AcrIIA5 mutants.

The IDR of AcrIIA5 played a key role in Cas9 inhibition, but only in the presence of the structured region. We investigated how the structured region of AcrIIA5 contributed to the Cas9 inhibition. The electrostatic surface potential representation of AcrIIA5 showed that positive and negative charges were densely clustered on opposite sides of the structure (Figure 5A). We selected those residues in well-defined secondary structures that constituted the positively or negatively charged surfaces, and replaced them with alanine for neutralization (Figure 5B and C). We first designed four positive-charge mutants, and assessed their inhibitory activity against Cas9 (Figure 5B). Apart from AcrIIA5_R115,K131_, which did not express as a soluble protein, AcrIIA5_K85_, AcrIIA5_K127,K137_ and AcrIIA5_K134,K136_ exhibited Cas9 inhibition comparable to that of wild-type AcrIIA5 (Figure 5D). We then examined the impact of the negatively charged surface of AcrIIA5 on Cas9 inhibition (Figure 5C). The AcrIIA5_E38,D41_, AcrIIA5_D93,E96_, AcrIIA5_D100,E104_ and AcrIIA5_E123,D125_ mutants employed in this study were fully competent for Cas9 inhibition, similar to wild-type AcrIIA5 (Figure 5E). Thus, perturbations of the positive- and negative-charge clusters did not significantly affect the inhibitory activity of AcrIIA5 against Cas9.

A recent phage plaquing study reported loss-of-function mutations of AcrIIA5 against Cas9 *in vivo* (41). Therein, the authors reported that AcrIIA5_D50,R62_ and AcrIIA5_D74,K85,K88_ mutants completely lost the inhibitory activity against various type II-A and type II-C Cas9 homologs, whereas AcrIIA5_H66,N70,H73_ failed to inhibit Cas9 homologs except for *S. pyogenes* Cas9. When the mutation sites were mapped onto the three-dimensional structure of AcrIIA5, they were mainly clustered near the long β3–β4 loop region (Figure 5F). Specifically, His66, Asn70, His73, and Asp74 were located in the β3–β4 loop region. Asp50, Arg62 and Lys88 were in β1–β2 loop, β3 strand, and β4–α2 loop, respectively, and their side chains were in close neighborhood with the β3–β4 loop (Figure 5F). We prepared alanine mutants of these residues to investigate whether they would affect Cas9–sgRNA binding and inhibition of the nuclease activity. We excluded Lys85 from the mutant design, since we already showed that it did not affect Cas9 inhibition. The AcrIIA5_D50,R62_ and AcrIIA5_D74,K88_ mutants failed to inhibit the Cas9 nuclease activity, which was consistent with the phage plaquing assay (Figure 5G). Unexpectedly, AcrIIA5_H66,N70,H73_ did not inhibit Cas9, either, though the plaquing assay indicated a full activity against *S. pyogenes* Cas9 *in vivo* (Figure 5G, 41). It is not clear what caused the discrepancy of Cas9 inhibition in vitro and in vivo. We note that all three mutants induced large super-shifts of Cas9–sgRNA in the gel shift assay (Figure 5H). Taken together, we conclude that these AcrIIA5 mutants formed complexes with Cas9–sgRNA, which did not culminated in Cas9 inhibition. We recall that the β3–β4 loop did not adopt a well-defined conformation in the solution structure and exhibited mobility from the heteronuclear NOE data (Figures 1D and 2B). Thus, the key residues of AcrIIA5 required for Cas9 inhibition are located at the mobile loop region in the middle of the compact fold, in addition to the N-terminal IDR. We speculate that the disordered tail and the mobile loop of AcrIIA5 together interfere with proper positioning of guide RNA in Cas9, preventing correct placement and cleavage of target DNA.

## DISCUSSION

Acr proteins are generally small in size with diverse sequences and structures, and mainly target the interference complexes of the host CRISPR–Cas system (44–46). AcrIIA5 is unique among known Acr proteins in that it contains a long unstructured tail region at the N-terminus. The 22-residue tail also possesses an amino acid composition biased toward disorder, including three lysine, four arginine, and four serine residues (47). The low sequence complexity, lack of a regular secondary structure, and intrinsic mobility collectively define the N-terminal tail as an IDR. Unexpectedly, the IDR was one of the main determinants for Cas9 binding and inhibition. IDRs are common in sequence spaces, and known to facilitate protein–protein and protein–nucleic acid interactions (48,49). Our study provides the first example that Acr proteins can exploit IDRs for Cas9 inhibition. Multiple sequence alignment of AcrIIA5 homologs indicates conserved motifs in the IDR, further supporting its functional significance (Supplementary Figure S4).

An IDR can be generally viewed as a structural ensemble that samples a wide range of conformational spaces in a rapid dynamic equilibrium (Figure 2B). The conformational plasticity of IDR may be linked to the broad inhibitory spectrum of AcrIIA5 against diverse Cas9 homologs. It has been reported that AcrIIA5 strongly inhibits most type II-A and II-C Cas9 homologs, and also moderately inhibits type II-B Cas9 (41,42). The flexible IDR may form interaction surfaces that adapt to different Cas9 targets, leading to promiscuous Cas9 binding and inhibition. Further, inhibition of the widely divergent Cas9 targets by AcrIIA5 raises a possibility of fuzzy complexes, where the IDR of AcrIIA5 remains disordered even in complex with Cas9 targets (50). A broad-spectrum Acr activity would be beneficial for phage survival in hosts harboring multiple types of Cas9 proteins. Indeed, *S. thermophilus* possessed two distinct Cas9 nucleases, St1Cas9 and St3Cas9, both of which were inactivated by AcrIIA5 (23).

We showed that AcrIIA5 associated with Cas9–sgRNA, but not with apo-Cas9. In addition, AcrIIA5 did not exhibit a ribonuclease activity *in vitro*, whereas co-expression of AcrIIA5 degraded anti-repeat, stem–loops 1 and 2 regions of sgRNA bound to *Neisseria meningitidis* Cas9 *in vivo*, owing to unknown endogenous ribonucleases (41). These observations suggest that AcrIIA5 associates with Cas9 surface involved in sgRNA binding, and interferes with the correct positioning of sgRNA upon Cas9. When the cleavage sites of in vivo sgRNA degradation are shown in the three-dimensional structure, they are close to the bridge helix (BH) and REC1 domain of *N. meningitidis* Cas9, and distal from the RuvC or HNH nuclease domains (Supplementary Figure S5). This raises a possibility that the BH and REC1 domain may form the binding interfaces for AcrIIA5. Since AcrIIA5 inhibited several II-A and II-C Cas9 homologs, the binding interfaces of Cas9 likely have conserved sequence motifs. Interestingly, multiple sequence alignments showed highly conserved motifs in BH and REC1, which was universal in all II-A and II-C Cas9 homologs, locating potential binding interfaces (Supplementary Figures S6 and S7). Recently, Song *et al.* reported that AcrIIA5 inhibited the nuclease activity of the RuvC domain, but not the HNH domain, of *S. pyogenes* Cas9 (42). It is intriguing how AcrIIA5 binding impacts on the Cas9–sgRNA interaction and inhibits only the non-target strand of substrate dsDNA. In our tentative binding model, AcrIIA5 binds to guide RNA binding region of Cas9, e.g. BH and REC1, and the N-terminal IDR may extend toward the RuvC nuclease domain for Cas9 inhibition.

In conclusion, we report that AcrIIA5 employs an IDR to inhibit Cas9, illustrating a novel Acr mechanism. The size and sequence of the IDR modulate AcrIIA5 binding to Cas9 and inhibition of the nuclease activity. We suppose that the dynamic nature of the IDR may play an important role in the broad-spectrum Cas9 inhibition by AcrIIA5. Our study expands the repertoire of structures and mechanisms involved in Acr function and introduces the IDR into the battlefield between phage and host bacteria.

## DATA AVAILABILITY

The atomic coordinates of the solution structure of AcrIIA5 and the NMR restraints have been deposited in the Protein Data Bank (PDB code 6LKF), and in the Biological Magnetic Resonance Bank (accession number 50185), respectively.

## Supplementary Material

gkaa512_Supplemental_File
