# Supplementary material for: Intrinsic disorder is essential for Cas9 inhibition of anti-CRISPR AcrIIA5
Source: Nucleic Acids Res. 2020 Jun 16;48(13):7584–94. doi: 10.1093/nar/gkaa512 (PMC7367191; doi:10.1093/nar/gkaa512)

Supplementary Figure S1. (A) The ^{1}H–^{15}N HSQC spectrum of 0.5 mM AcrIIA5 in 10 mM sodium phosphate, pH 7.0, and 500 mM NaCl at 25°C. The backbone amide resonances are annotated with the residue types and numbers. The inset at top left is a magnified view of the central portion of the spectrum, indicated by the rectangle. (B) Superimposed HSQC spectra of ^{15}N-AcrIIA5 (*black*) and ^{15}N-AcrIIA5$_{\Delta 20}$ (*red*).

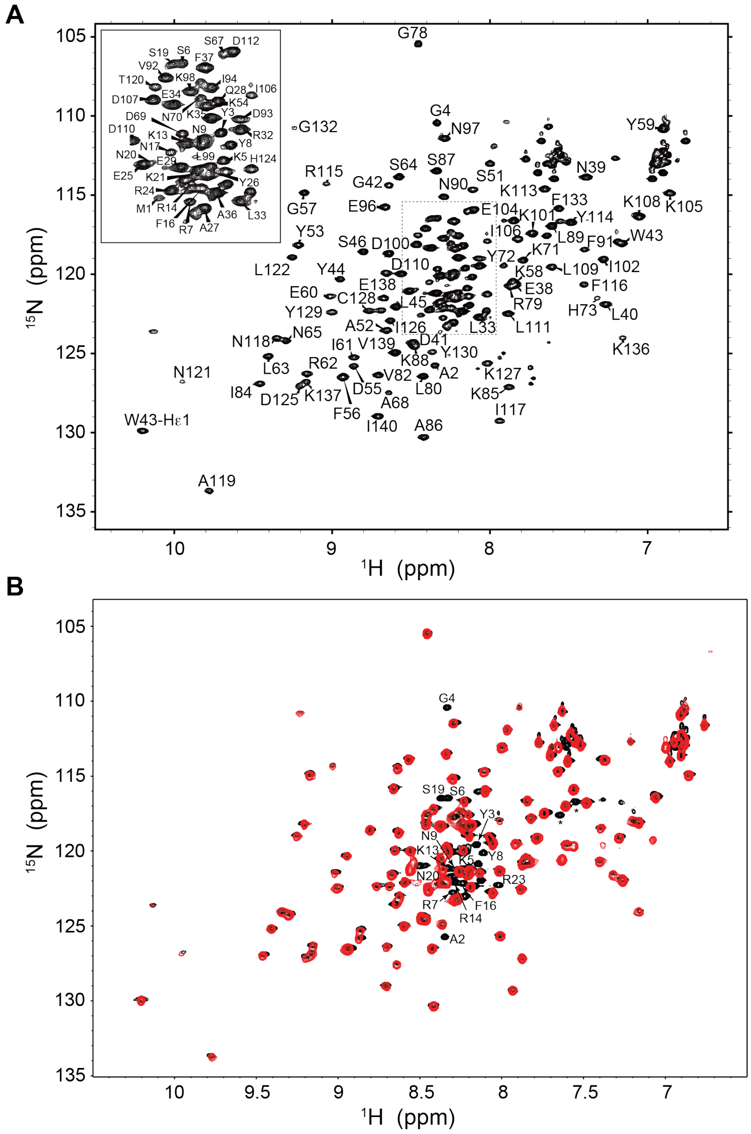

Supplementary Figure S2. Inhibition of Cas12a and Cas9 by AcrIIA5, and Cas9 stability in the presence of AcrIIA5 and mutants. (A) DNA cleavage assay of 0.2 µM *L. bacterium* Cas12a−sgRNA with 3 µM AcrIIA5, and (B) gel shift assay of 0.2 µM Cas12a−sgRNA with AcrIIA5 (0.4, 0.8, 2, and 4 µM). The reaction buffer was the same as that used for Cas9. DNA cleavage assay of *S. pyogenes* Cas9−sgRNA (0.5 µM) in the presence of (C) AcrIIA5$_{\Delta 20}$ mixed with the IDR peptide in a 1:1 ratio (0.25, 0.5, 1, and 3 µM), and (D) AcrIIA5$_{RKR}$ mixed with the IDR peptide in a 1:1 ratio (0.25, 0.5, 1, and 3 µM). Stability test of Cas9 in the presence of AcrIIA5 at (E) varying ionic strengths at 4°C after 16 h incubation, and (F) varying temperatures after 1 h incubation. (G) DNA cleavage assay of Cas9−sgRNA with AcrIIA5. The asterisk sign of AcrIIA5 in the last lane denotes that AcrIIA5 was removed from the inhibition reaction of Cas9–sgRNA.

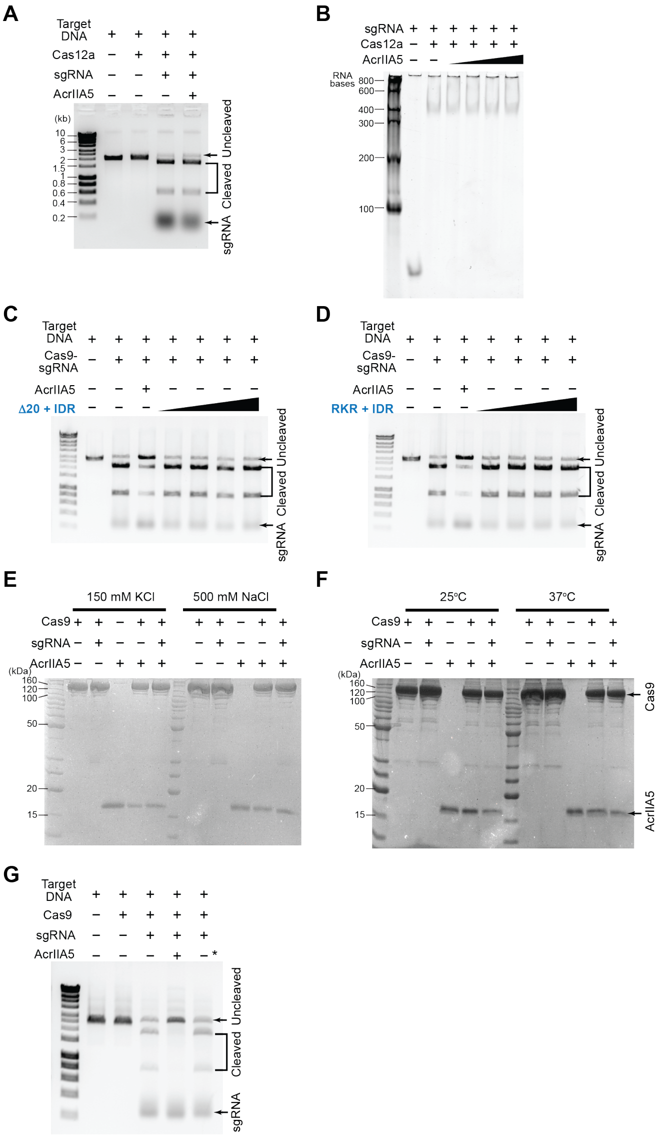

Supplementary Figure S3. 2-D ^{1}H–^{15}N HSQC NMR spectra of AcrIIA5 in the absence (*black*) and the presence (*green*) of apo-Cas9.

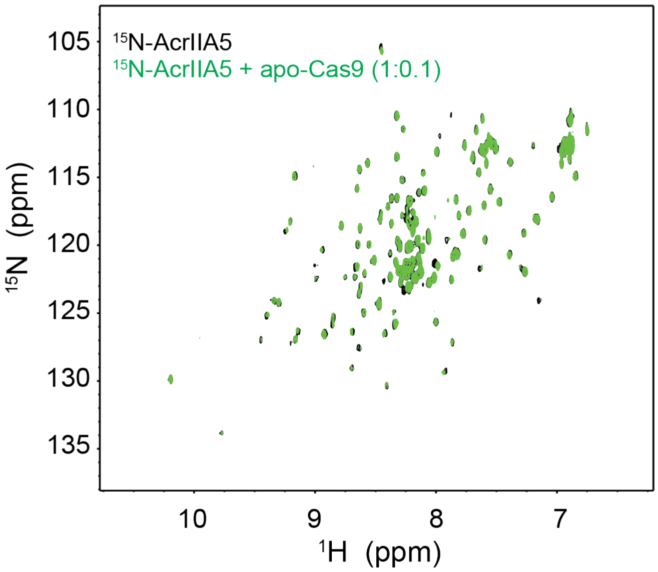

Supplementary Figure S4. Multiple sequence alignment of AcrIIA5 and its homologs. Conserved and variable sequences among AcrIIA5 homologs. The sequences were aligned using the Clustal Omega program and colored using the ESPript 3.0 program. AcrIIA5 used in this study has the amino acid sequence of the GenBank ID ASD50988.1. Secondary structures are shown schematically above the alignment. Basic residues in the IDR are indicated with filled squares in *blue* under the alignment. Acidic and basic residues in the structured region selected for mutagenesis are indicated with filled circles in *red* and *blue*, respectively, and residues previously shown to be involved in Cas9 inhibition (41) are indicated by *red* asterisks under the alignment.

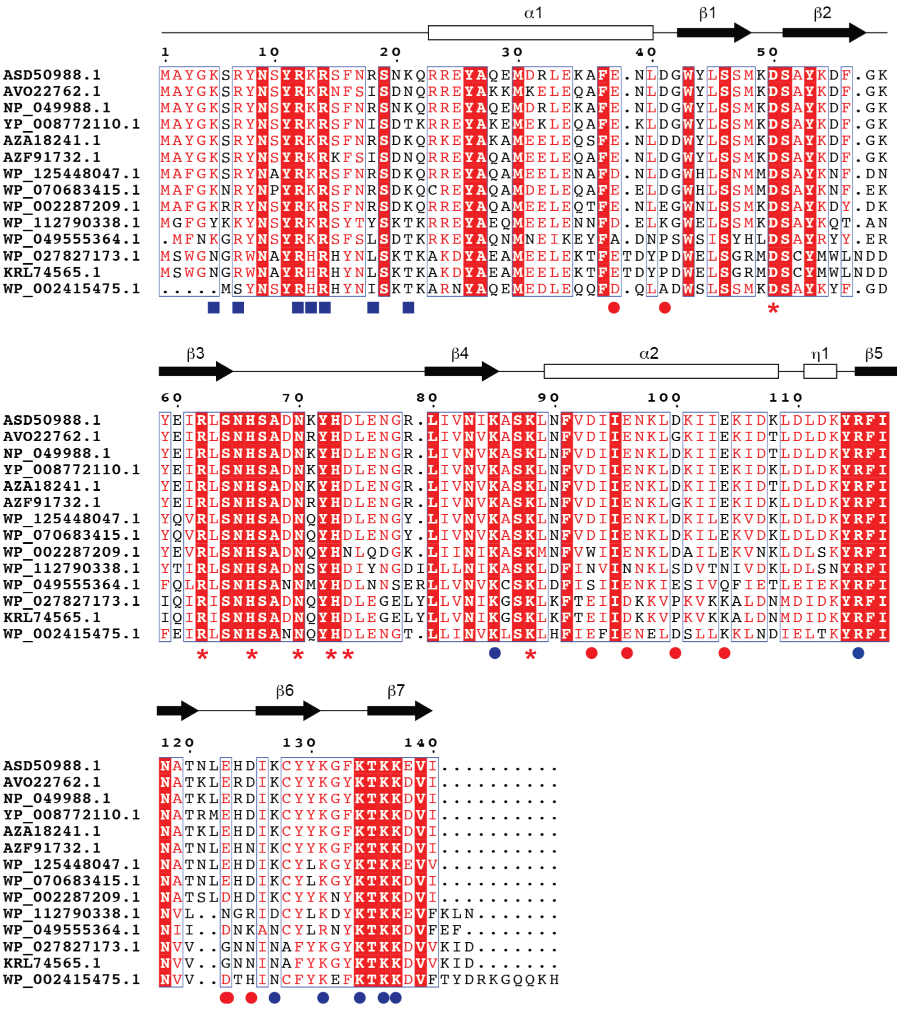

Supplementary Figure S5. The domain structures of *N. meningitidis* Cas9 are annotated with the residue numbers (*top*). The three-dimensional structures of *N. meningitidis* Cas9–sgRNA are shown in a ribbon diagram with the same color code (PDB code 6JDQ, *bottom*). The *light blue* ribbon represents sgRNA, and the magenta spheres denote the sgRNA degradation sites: G57, U59, C73, C87, and G108. Those residues highly conserved among II-A and II-C Cas9 homologs are shown as a space-filling model, and colored in *cyan* (bridge helix) and in *salmon* (REC1 domain). BH: bridge helix; WED: wedge domain; PI: PAM interacting domain.

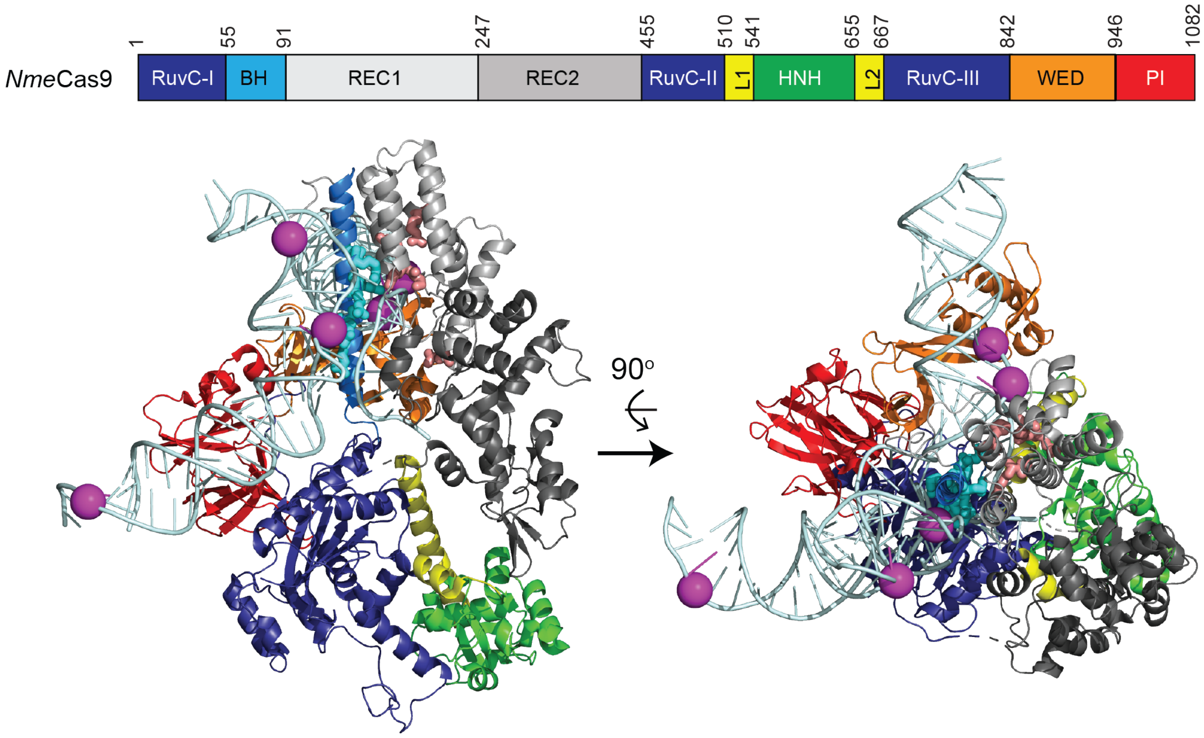

Supplementary Figure S6. Multiple sequence alignment of type II-A Cas9 homologs. The domain structures of *S. pyogenes* Cas9 (*Spy*Cas9) are shown above the alignment. *Spy*: *Streptococcus pyogenes* (WP_032464890), *Sth*: *Streptococcus thermophilus* (WP_082309079.1), *Sau*: *Staphylococcus aureus* (J7RUA5.1). Residues exclusively conserved among II-A and II-C Cas9 homologs are annotated with *red* asterisks. RuvC: RuvC nuclease domain; BH: bridge helix; REC: recognition domain; HNH: HNH nuclease domain; TOPO: TOPO-homology domain; CTD: C-terminal PAM interacting domain.

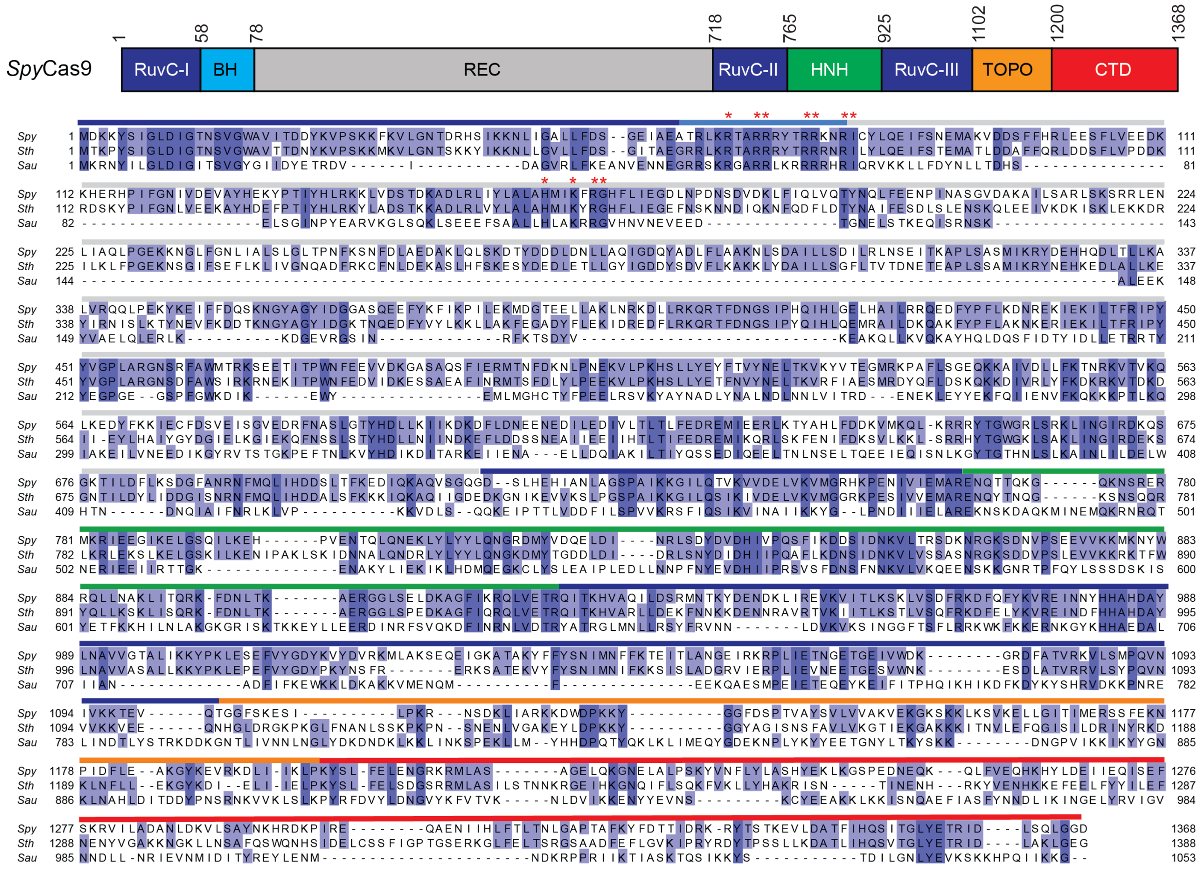

Supplementary Figure S7. Multiple sequence alignment of type II-C Cas9 homologs. The domain structures of *Neisseria meningitidis* Cas9 (*Nme*Cas9) are shown above the alignment. *Nme*: *Neisseria meningitidis* (WP_061704949.1), *Hpa*: *Haemophilus parainfluenzae* (WP_115180630.1), *Boe*: *Brackiella oedipodis* (WP_051532411.1), *Geo*: *Geobacillus stearothermophilus* (RLP87161.1), *Kla*: *Kiloniella laminariae* (WP_020594047.1), *Cdi*: *Corynebacterium diphtheriae* (VVH30411.1), *Cje*: *Campylobacter jejuni* (PVX13058.1). Residues exclusively conserved among II-C and II-A Cas9 homologs are annotated with *red* asterisks. RuvC: RuvC nuclease domain; BH: bridge helix; REC: recognition domain; WED: wedge domain; PI: PAM interacting domain.

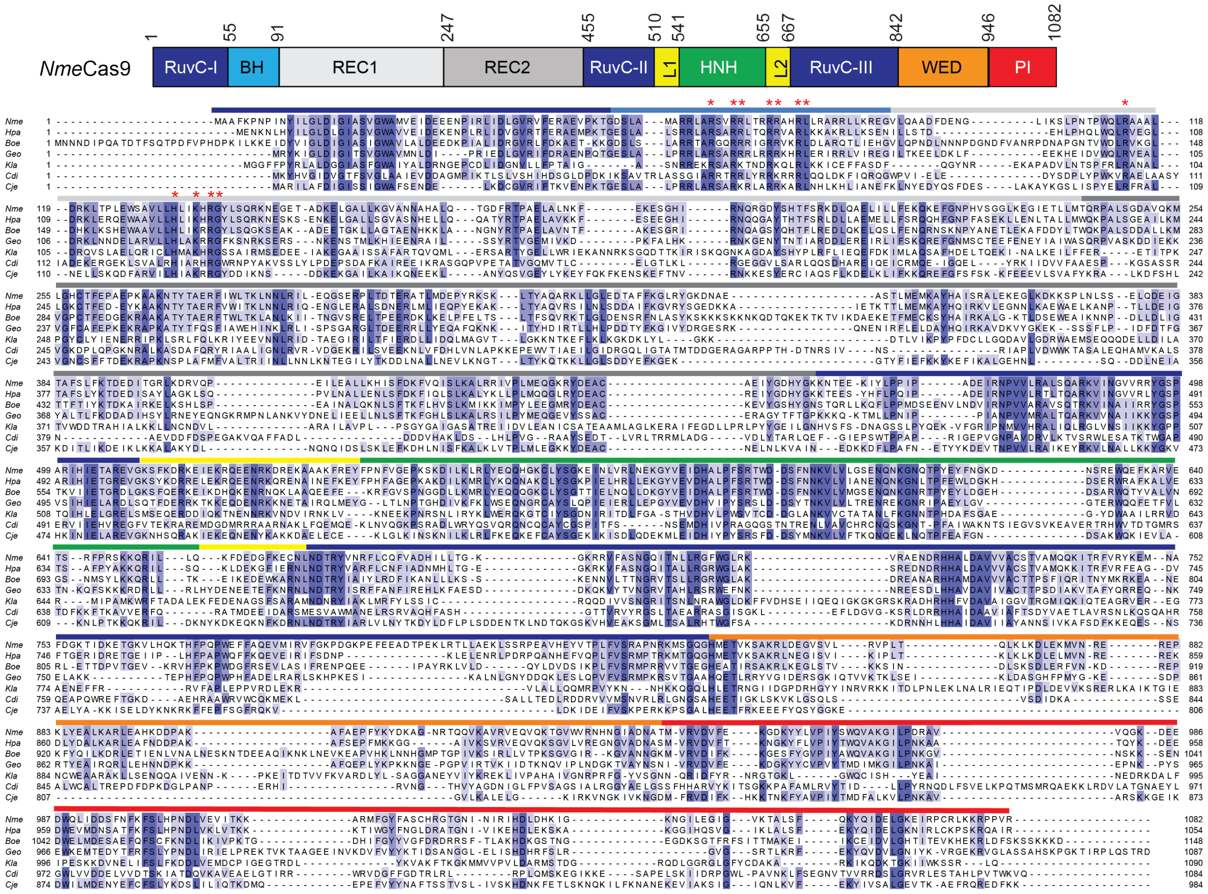

Supplement: gkaa512_Supplemental_File [file gkaa512_supplemental_file.pdf]
